# What is the appropriate gravel size during ureteroscopy lithotripsy? An in vitro evaluation

**DOI:** 10.1007/s00240-023-01430-w

**Published:** 2023-03-16

**Authors:** Baiyang Song, Dan Jin, Yue Cheng, Zhengyi Wang, Fengqi Wang, Li Fang

**Affiliations:** 1 School of Medicine, Ningbo University, Ningbo, 315211 Zhejiang People’s Republic of China; 2 Department of Urology, The First Affiliated Hospital of Ningbo University, 59 Liuting Road, Ningbo, 315010 Zhejiang People’s Republic of China; 3 Department of Urology, Shangyu People’s Hospital, Shaoxing, 312300 Zhejiang People’s Republic of China; 4 Ningbo Clinical Research Center for Urological Disease, Ningbo, 315010 Zhejiang People’s Republic of China

**Keywords:** Urolithiasis, Gravel size, Intraoperative calculi excretion, Flexible ureteroscopy

## Abstract

To propose the suitable diameter of calculus debris produced during flexible ureteroscopy lithotripsy (fURL). A glass tube was used to simulate the stone excretion process during Furl. Different stone diameters (0.50–1.00 mm, 0.25–0.50 mm, and 0.10–0.25 mm) with three sizes of flexible ureteroscopy (fURS) (7.5Fr, 8.7Fr, and 9.9Fr) and ureteral access sheath (UAS) (12/14Fr) with or without negative pressure suction were employed in the experiment. The intraoperative calculi excretion (ICE) was recorded according to the stones discharged from the gap between fURS and UAS. The ICE raised significantly in thinner fURS and UAS due to the smaller Ratio of Endoscope-Sheath Diameter (RESD). The gravel size ≤ 0.25 mm was conducive to drainage with traditional UAS, while using fURS with negative-pressure UAS could significantly improve ICE. The gravel size ≤ 0.5 mm was conducive to expulsion. We clarify that ICE during ureteroscopy relates to RESD and negative pressure suction. The proper size of the stone fragment is critical in ensuring the expulsion during fURL, ≤ 0.25 mm in traditional UAS and ≤ 0.50 mm in negative-pressure UAS, respectively.

## Introduction

Urinary calculus is a common urological disease usually managed by lithotripsy [1]. Minimally invasive surgeries have become pivotal in treatment, with flexible ureteroscopy lithotripsy (fURL) having abundantly replaced open surgery over the past decades [2]. As a classical approach, fURL is just used to break stones into fragments, and the resulting stone residue needs to be vented by itself [2–4]. Many patients cannot discharge the stones successfully after the operation due to the gravel size, location, ureteral stricture, and other factors, resulting in renal colic, stone street formation, and other complications [5, 6]. The active extraction of stone fragments using a stone basket can be cumbersome and time-consuming [7–9]. Therefore, allowing the fragments to be discharged during the operation is an effective method.

Stones must be fragmented into smaller gravels before evacuation due to the intrinsic size limitations of fURS access routes [4, 8]. The smaller gravel size and the larger gap between fURS and ureteral access sheath (UAS) are known as the Ratio of Endoscope-Sheath Diameter (RESD) facilitate intraoperative calculi excretion (ICE) and stone-free rate (SFR) [10, 11]. Additionally, vacuum-assisted UAS can actively control the intrarenal pressure (IRP) and promote ICE [12].

The smaller gravel size improves ICE and SFR during fURL; it may lead to longer operation time and a higher rate of postoperative complications like fever and infection [13–15]. Exploring an appropriate gravel size can reduce the operation time and postoperative complications while controlling SFR. The present study employs a glass tube to simulate the excretion of gravels during fURL in vitro. We focus on the condition of various RESDs with or without negative-pressure UAS to explore the appropriate gravel size to guide the clinical work.

## Materials and methods

### Materials

**Fig. 1:**
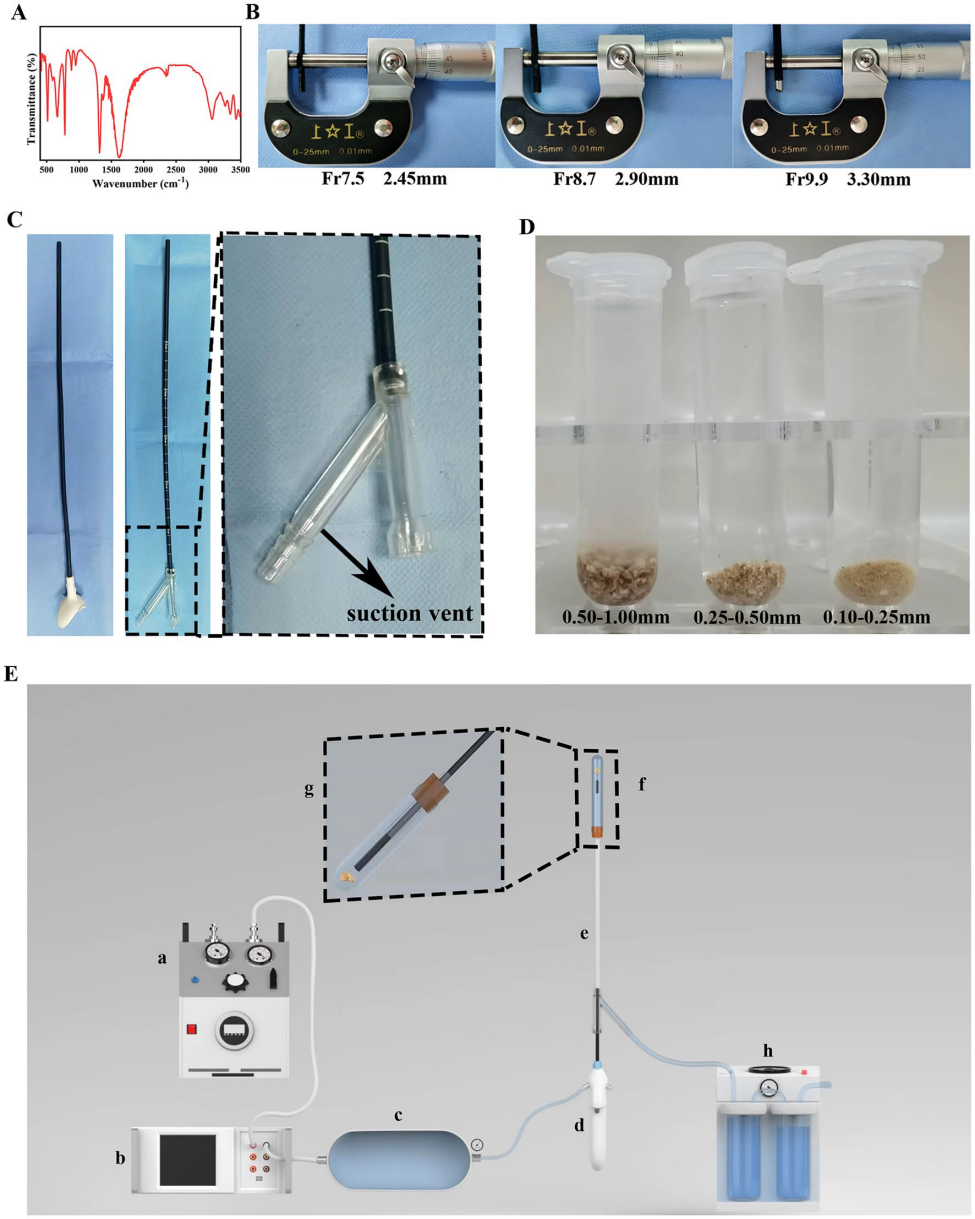
Materials and equipment for the experiment. **A** FTIR spectra of calcium oxalate stones. **B** Out diameter of different flexible ureteroscopy. **C** Diagram of traditional and negative-pressure UAS. **D** Stones of different diameters. **E** Instrument connection diagram. **a** electric barostat, **b** automatic pressure controller, **c** water storage tank, **d** flexible ureteroscopy, **e** UAS, **f** glass tube and stones, **g** magnification of **f**, **h** negative pressure suction device

Calcium oxalate stones were collected from patients undergoing percutaneous nephrolithotomy (PCNL) and tested using a Fourier Infrared Spectrometer in this study (Fig. 1A). Following devices were used: three fURSs with different diameters (7.5Fr, Pusen, Zhuhai, China; 8.7Fr, Anqing, Shanghai, China; and 9.9Fr, Woek, Nanchang, China) (Fig. 1B), the traditional UAS (T-UAS) (12/14Fr, 35 cm, Zhexin, Hangzhou, China), the negative-pressure UAS (NP-UAS) (12/14Fr, 35 cm, Madewell, Haiyan, China) (Fig. 1C), a negative pressure suction device (Yuyue, A7-23D, Nanjing, China), and an automatic pressure controller and electric barostat (Puli, Beijing, China).

### In vitro experiments

The stones were divided into three groups (0.50–1.00 mm, 0.25–0.50 mm, and 0.10–0.25 mm) using stainless steel screening (Fig. 1D). First, we screened the stones with a 1.00 mm aperture screening to obtain stones ≤ 1.00 mm. Subsequently, the stones were passed through a 0.50 mm screening to obtain stones of 0.50 mm–1.00 mm. Similarly, 0.25 mm–0.50 mm and 0.10 mm–0.25 mm stones were obtained by using 0.25 mm and 0.10 mm screening, respectively. Due to the irregular shape of the stones, there may be cases where the stone length or diameter is larger than the calibrated value, but in general, the stone size is within the calibrated interval.

The automatic constant flow pump was connected to the UASs, and the flow rate was set to 100 mL/min. After 10 s of stabilization, the pressure of UAS water inlets was measured to be 35 KPa (7.5Fr), 35 KPa (8.7Fr), and 36 KPa (9.9Fr).

Infusion-suction instruments were connected (Fig. 1E). The electric barostat (a) was connected to the automatic pressure controller (b), the pressure of (a) was set to 50 kPa, and the measured inlet water pressure of fURSs was input into (b) to ensure that the water storage tank (c) output flow rate was 100 mL/min. The fURS was connected to the water outlet and entered the kidney model via the UAS to simulate the stone removal during surgery. The negative pressure suction device was connected to the suction channel on the UAS.

Stones weighing 5 g were placed in the glass tube, followed by inserting the UAS and fURS into the tube and starting equipment. After 30 s, the stones discharged from the tube were collected and dried thoroughly. Finally, the different-sized stones were weighted. The above experiments were repeated five times in each group with different RESD. The definition of RESD is based on our previous study [11], i.e. the ratio of outer diameter of fURSs and inner diameter of UASs.

### Statistical analysis

The data were processed using SPSS version 26.0. All data were expressed as mean ± standard deviation (SD). Hypotheses regarding differences among the values were compared using the Student’s *t*-test, Mann Whitney rank sum test, or one-way ANOVA test, with *p* < 0.05 considered statistically significant.

## Results

**Fig. 2:**
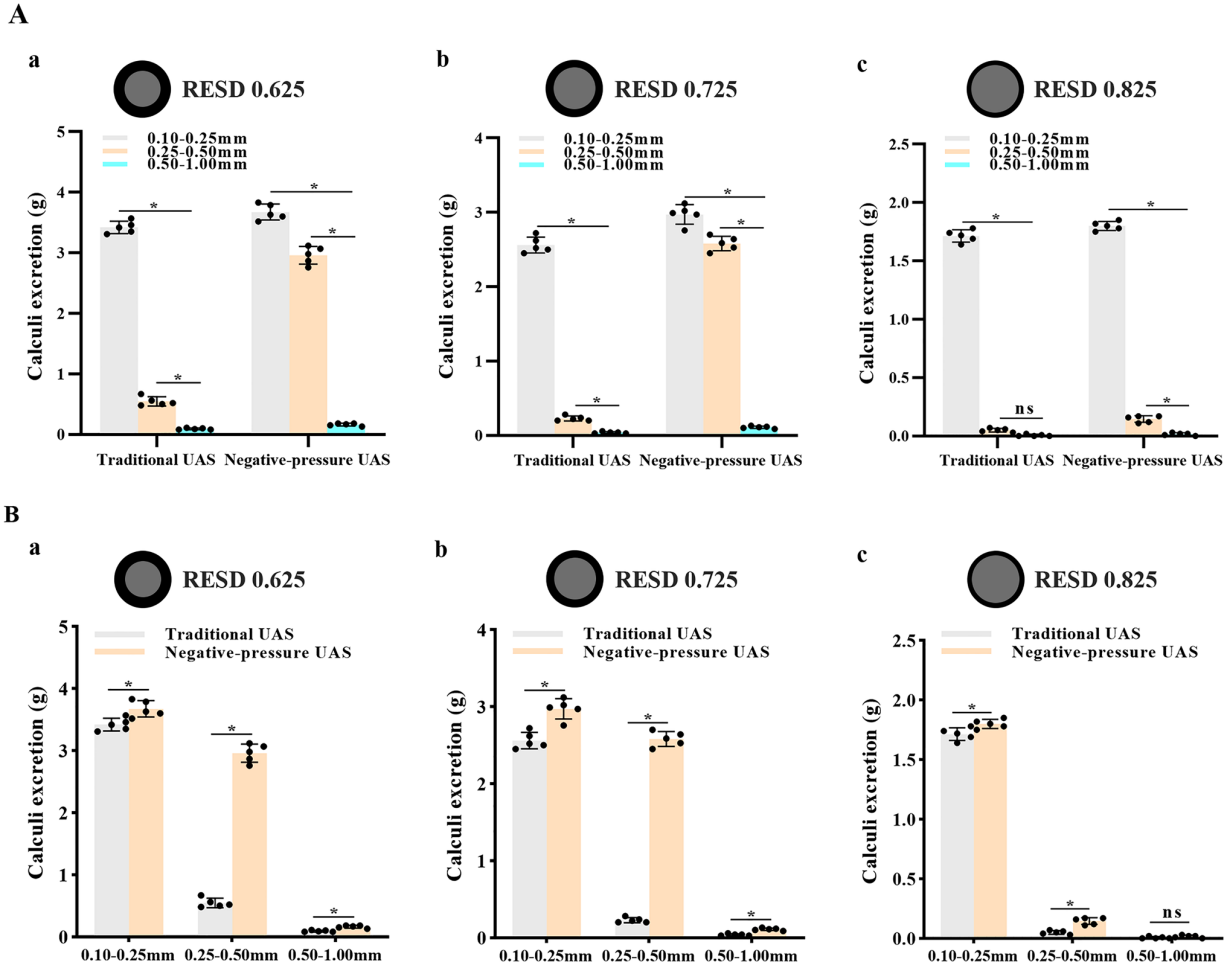
Results of intraoperative calculi excretion comparison. **A** Comparison of calculi excretion for different gravel sizes, a, b, and c refer to fURS of Fr7.5, Fr8.7, and Fr9.9. **B** Comparison of calculi excretion for T-UAS and NP-UAS, a, b, and c refer to fURS of Fr7.5, Fr8.7, and Fr9.9. All data are presented as mean ± SD (*p < 0.05; ns: *p* > 0.05)

Figure 2 demonstrates that when the RESD is 0.625, whether T-UAS or NP-UAS is used under the same stone size, the stone excretion is greater than that of the RESD groups of 0.725 and 0.825, while the RESD group of 0.825 has the least calculi excretion. Consequently, the smaller the RESD, the larger the gap between fURS and UAS, and the easier it is for stone powders to be discharged through the gap during operation.

When the RESD is 0.625 or 0.725, it is evident that the calculi excretion increases as gravel size decreases, regardless of the UAS employed (Fig. 2Aa and Ab). When the RESD is 0.825, the amount of calculi excretion in the NP-UAS group increases with the decrease in gravel size. The T-UAS group displays similar results of calculi excretion with the gravel size from 0.25 to 1.00 mm (Fig. 2Ac). Besides, the NP-UAS can significantly improve the calculi excretion with the RESD of 0.625 or 0.725 (Fig. 2Ba and Bb). With the RESD of 0.825, the NP-UAS promotes the stone excretion with a diameter from 0.10 to 0.50 mm, while it provides poor results of calculi excretion, similar to the T-UAS (Fig. 2Bc).

## Discussion

The present study investigated the appropriate gravel size during fURL, achieving an excellent ICE result. We demonstrated that NP-UAS promoted ICE compared to T-UAS with the RESD < 0.75. Furthermore, we demonstrated that the ideal gravel sizes differed, ≤ 0.25 mm and ≤ 0.50 mm in T-UAS and NP-UAS, respectively, which provides a theoretical basis for our later development of intelligent lithotripsy system.

When using NP-UAS, a high flow rate is required due to the negative-pressure suction. Otherwise, it may lead to a rapid intraoperative aspiration of the water without achieving the effect of suctioning the gravels. In order to match the flow rate of the NP-UAS clinically, we set it to 100 mL/min. Our previous research demonstrated that the RESD < 0.75 guarantees a safe IRP during the fURL process [11]. This study employed 12/14 UAS as the fURS access route. The RESD reached 0.825 when we chose the Fr9.9 fURS, making it impossible to ensure a safe IRP intraoperatively. However, decreasing the gap between fURS and UAS results in a substantially lower ICE, especially when applying a T-UAS (Fig. 2Ac and Bc). When we employed fURS of Fr7.5 and Fr8.5, RESDs were 0.625 and 0.725, respectively. They guaranteed a solid IRP, considerably improving the ICE. Figure 1Aa–Ab suggest that the smaller the gravel size, the higher the ICE. The same fUAS revealed a dramatic decrease in ICE using the T-UAS. Comparing 0.25–0.50 mm gravels to 0.10–0.25 mm gravels, 0.25 mm was a critical value for treatment. However, the critical value was 0.50 mm using NP-UAS, illustrating that NP-UAS broadened the range of appropriate gravel size during fURL. Figures 2Ba–Bb demonstrate that the difference in ICE between NP-UAS and T-UAS groups was significantly greater for gravels of 0.10–0.25 mm than for gravels of 0.25–0.50 mm. Therefore, we consider that the appropriate gravel size is ≤ 0.50 mm for fURS combined with NP-UAS for lithotripsy and ≤ 0.25 mm for combined T-UAS at appropriate RESD (< 0.75).

Dusting lithotripsy facilitates intraoperative and postoperative stone expulsion and improves SFR [4, 16, 17]. According to the latest definition of stone dust proposed by Keller et al*.*, stone particles must fulfill the following criteria: stones float spontaneously under 40 cm H_2_O perfusion pressure, the average settling time in 10 cm salt solution is > 2 s, and they can be pumped through the 3.6Fr working channel. Ultimately, the size of ≤ 0.25 mm gravels better met the requirements [13]. Our preliminary experiments revealed that gravels < 0.1 mm were difficult to precipitate and collect. Thus, the stone sizes studied were positioned in groups of 0.10–0.25 mm, 0.25–0.50 mm, and 0.50–1.00 mm. High ICE was achieved for stones ≤ 0.25 mm when the T-UAS was used, consistent with literature reports [13]. Nevertheless, NP-UAS can broaden the range of stone dust to some extent. We believe that the stone dust of fURS combined with NP-UAS for fURL is defined as ≤ 0.50 mm at an appropriate RESD (< 0.75).

NP-UAS can prevent excessive intraoperative IRP and lower the risk of postoperative infection and fever by converting passive water circulation to active under negative pressure [12, 18–21]. Simultaneously, active stone suction aid in the release of gravels from the gap between fURS and UAS, decreasing the "snow storm" phenomena during dusting lithotripsy and preserving intraoperative visibility [13]. The greatest strength of this study is that NP-UAS has the potential to broaden the appropriate gravel size, thereby improving ICE and decreasing the incidence of postoperative complications. However, the 12/14Fr UAS cannot be inserted in some patients because of their thin ureters. For them, a stent can be left preoperatively or a thinner UAS can be used on the condition of RESD < 0.75.

This study is a pre-study for subsequent experiments to initially verify the appropriate gravel size during fURL. The glass tube is used as the experimental model because it is easily accessible, reproducible, and does not collapse during negative pressure suction. Besides the fact that a glass tube is not synonymous with a normal kidney, there may be additional factors affecting ICE. Therefore, our next steps are to design an appropriate kidney model or in vivo experiments and insert the fiber to fURS to identify other parameters influencing ICE, with the ultimate goal of guiding the development of medical instruments and clinical work.

## Conclusion

In conclusion, we discovered that the appropriate gravel size is ≤ 0.25 mm with traditional UAS and ≤ 0.50 mm with negative-pressure UAS, respectively. Clinically, we recommend choosing negative-pressure UAS and fragmenting stones smaller than 0.50 mm wherever possible while attempting to keep RESD < 0.75 as low as is safe for surgery.

## Data Availability

All data analysed during this study are included in this published article figures and the datasets analysed during the current study are available from the corresponding author on reasonable request.
